# Management of Patients with Left Ventricular Assist Device during the COVID-19 Pandemic

**DOI:** 10.3390/medicina58010116

**Published:** 2022-01-13

**Authors:** Gassan Moady, Tuvia Ben Gal, Shaul Atar

**Affiliations:** 1 Department of Cardiology, Galilee Medical Center, 1 Ben Tzvi Blvd., Nahariya 2210001, Israel; shaul.atar@gmail.com; 2 Azrieli Faculty of Medicine, Bar Ilan University, Safed 1311502, Israel; 3 Department of Cardiology, Rabin Medical Center, Petah Tikva 4941492, Israel; bengalt@clalit.org.il; 4 Sackler Faculty of Medicine, Tel Aviv University, Tel Aviv 6139001, Israel

**Keywords:** coronavirus disease, ventricular assist device, heart failure

## Abstract

The novel coronavirus disease 2019 (COVID-19) is an infectious disease with multi-organ involvement, including the cardiovascular system. The disease may cause several cardiovascular complications, and may increase morbidity and mortality among patients with background cardiovascular disease. Patients with advanced heart failure are often treated with left ventricular assist device (LVAD), and represent a unique population mandating multi-disciplinary approach. Several aspects of COVID-19 should be taken into account in LVAD implants, including right ventricular involvement, hemodynamic alterations, thromboembolic and haemorrhagic complications, and the psychological effects of social isolation. Patients with VAD and suspected COVID-19 should be transferred to specialized centers for better management of complications. Here, we review the implications of COVID-19 pandemic on LVAD patients with our recommendations for appropriate management.

## 1. Introduction

Despite improvements in pharmacological and invasive therapies in heart failure, some patients still suffer from intractable disease with a need for advanced mechanical support or heart transplantation. Left ventricular assist device (LVAD) is associated with improved survival among patients with advanced heart failure and may be used as a bridge to transplantation or as a destination therapy [1,2]. Implanted patients need to be managed by expert staff of clinicians, nurses, and coordinators. The novel coronavirus disease 2019 (COVID-19) caused by the severe acute respiratory syndrome 2 virus (SARS-CoV-2) is associated with several cardiovascular complications and may increase morbidity and mortality among patients with background heart disease [3]. Some of the reported COVID-19 related cardiovascular complications are myocardial injury, myocarditis, arrhythmia, pulmonary embolism, and takotsubo syndrome [4,5,6]. Similar to other cardiovascular patients, LVAD implants are at increased risk for complications during the acute infection. Moreover, several considerations need to be taken into account while managing this unique population since hemodynamic and hematologic alterations that have been described in COVID-19 may critically affect LVAD implants. Infection with SARS-CoV-2 is associated with several cardiovascular and hematologic changes that may result in drop in LVAD speed, low flow and suction events, and lower pulsatility index. The treatment of LVAD patients mandates multi-disciplinary approach with experienced physicians, nurses and LVAD coordinators, given that close monitoring of clinical condition, device parameters, and laboratory results are the cornerstones of the treatment. Several cautions are needed in order to minimize the risk of infection among patients, and meanwhile to keep adequate medical response to them. Here, we summarize the effects of the current COVID-19 pandemic on LVAD patients.

## 2. Outcomes of LVAD Patients with COVID-19

There are several reasons for increased susceptibility of LVAD patients for COVID-19 infection. Among these reasons: the demographics of the patients (elderly with several comorbidities), abnormal immune system, and a variety of cytokines activation [7]. With the onset of the COVID-19 pandemic, cases of acute SARS-CoV-2 infection have been reported in LVAD implants [8,9,10,11,12,13,14,15,16,17,18,19,20,21]. In one series of 28 LVAD implants with confirmed COVID-19, 79% were male with median age 65 years, and the majority (82%) were destination therapy (11). Twenty-four (86%) of the patients in this series were hospitalized, while four (14%) received out of hospital management. The implanted devices were HeartMate2 (HM2) (21%), HeartWare^TM^, HVAD^TM^ (36%), and HeartMate3 (HM3) (43%). Mechanical ventilation was required in five (18%) patients, and the mortality rate was 32% from COVID-19 complications during the index hospitalization and after a median duration of 80 days. In another registry of 80 patients, 60% required hospitalization, and eight patients (20%) died, often after lengthy hospitalizations (13). The high mortality rate may be explained by the underlying cardiac condition of the patients along with the hemodynamic and hematologic changes that directly affect device function. Acute respiratory failure secondary to ARDS should be managed similar to other patients, with the exception that prone position may have some undesirable effects in LVAD implants (discussed later).

## 3. Diagnostic Challenges

Patients with heart failure often suffer from dyspnea and cough during exacerbations, symptoms that may be indistinguishable from those of COVID-19 [22]. The overlap in the presenting symptoms may subsequently cause a delay in accurate diagnosis of COVID-19. Moreover, biomarkers such as natriuretic peptides are not reliable for distinguishing congestion from COVID-19 since their levels are elevated in both conditions. Lactate dehydrogenase (LDH) level is useful as a marker of lung injury and as a prognostic marker in patients with ARDS [23]. In LVAD patients, LDH levels should be interpreted cautiously since high levels may indicate pump thrombosis [24]. Although the incidence of pump thrombosis is relatively low in the current most-commonly used devices (HM3), altered thrombotic state in COVID-19 may affect the incidence of this complication during the acute and chronic phase of the disease.

## 4. Complications

### 4.1. Right Ventricular Failure

One of the reported cardiovascular complications of COVID-19 is acute right ventricular (RV) dysfunction. According to one review, the prevalence of RV dysfunction was estimated to be approximately 20% with high grade of heterogeneity and increased risk in severe disease [25]. Acute RV failure may be encountered in COVID-19 as part of biventricular failure or as an isolated abnormality. Potential mechanisms for biventricular failure include myocarditis or global ischemia secondary to sepsis, while isolated RV failure may be the consequence of pulmonary embolism, pulmonary disease, pneumonia, or ARDS due to direct lung injury from the viral infection [25,26]. Regardless of the underlying etiology, RV dysfunction is associated with deleterious effects in LVAD implants with subsequent increased short and long-term morbidity and mortality since they have no RV support (unless they have biventricular device).

### 4.2. Hematologic Changes

Multiple mechanisms contribute to the abnormal coagulation profile in patients with COVID-19. The spectrum of COVID-19 related hematologic manifestations vary from abnormal laboratory tests to venous and arterial thromboembolic events and severe disseminated intravascular coagulation (DIC) [27,28]. Prolongation of prothrombin time (PT) and activated partial thromboplastin time (aPTT) with mild thrombocytopenia are commonly encountered in hospitalized patients that may progress to DIC in severe rare cases [28]. Endothelial dysfunction mediated by oxidative stress, activation of thrombotic factors such as von Willebrand factor and complement system, and dysregulation of immune response as part of the cytokine storm may all contribute to the high incidence of thrombotic events observed in COVID-19 despite the administration of anticoagulant prophylaxis in critically ill patients. The markedly activated inflammatory process in the lung together with pulmonary endothelial dysfunction may explain the higher incidence of pulmonary embolism in COVID-19. Of note, bleeding manifestations even in the setting of DIC have not been commonly reported in COVID-19 [28]. Antithrombotic management of LVAD implants is based on anticoagulation with Vitamin K antagonists and antiplatelets with targeted strict therapeutic range of INR depending on patient characteristics and device type. Given the abovementioned abnormalities in COVID-19, strict monitoring of coagulation profile is highly recommended in LVAD patients during acute infection of COVID-19. Oral anticoagulation should be replaced by parental heparin during the acute phase of labile status, and heparin therapy should be guided by anti-Xa levels (targeting between 0.5 and 0.7 IU/mL) rather than aPTT, since COVID-19 patients commonly have circulatory antibodies, which may disturb aPTT [19]. Special attention should be given to high-risk patients and among those with high D-dimer and low fibrinogen levels [9].

## 5. General Considerations during the Acute Infection

For patients with mild symptoms of COVID-19, home isolation with close surveillance is advised, while patients with moderate or severe disease should be admitted to intensive care units for possible hemodynamic or respiratory support. Close monitoring of hemodynamic and device parameters along with tests for blood count, anti-Xa, D-dimer, fibrinogen and N-terminal-pro hormone brain natriuretic peptide (NT-proBNP) levels are needed. Monitoring of device-flow parameters is essential for assessing the hemodynamic state of critically ill patients. A handheld Point-of-Care Ultrasound (POCUS) to assess septal position, RV function and the presence of pericardial effusion is preferred over complete echocardiography in order to minimize contact with infected patients. In general, the use of the prone position is recommended to treat refractory hypoxemia in ARDS. However, there is no sufficient data regarding the use of this technique in LVAD patients, and several fears have been taken into account, making it relatively contraindicated [19]. Venous return is compromised in the prone position and may further worsen RV function. In addition, compression of the outflow graft with subsequent flow reduction, and driveline site compression may also be a consequence of this position. In selected cases, the use of venous-venous extracorporeal membranous oxygenation (ECMO) may improve outcomes [29].

## 6. Management of Patients during Quarantine

Quarantine regime has been used worldwide to minimize disease spread. This regime, although useful in pandemic restriction, has several psychological and medical implications [30]. Patients may be concerned of being infected or fear that their caregiver may be either infected or isolated. In one study, patients with heart failure reported psychological distress, decreased physical activity, weight gain, and reduced adherence to medication and diet during lockdown [31]. In one case report, a patient with LVAD as destination therapy presented to the emergency room after a suicide attempt by drug overdose after feeling overwhelmed by stress during the lockdown [32].

**Figure 1:**
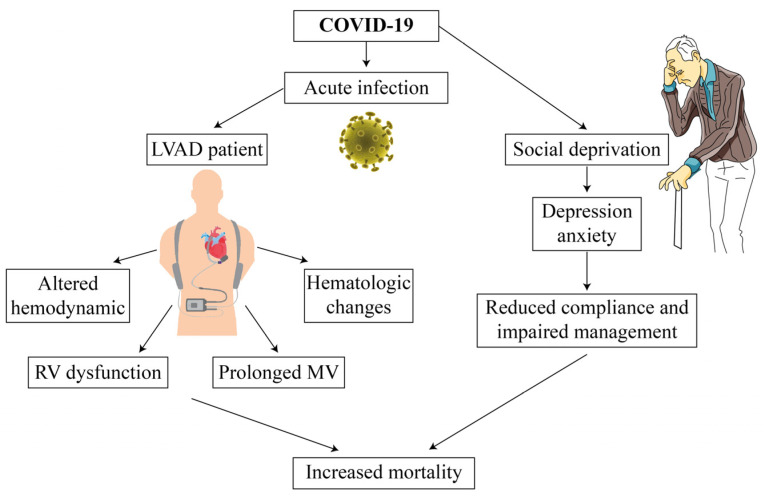
The Potential Implications of COVID-19 on LVAD Patients.

Admission to hospital for LVAD implants during the current pandemic should be minimized to reduce the risk of contact with infected patients. Due to these restrictions, close monitoring of clinical status, device function and routine blood tests may be not optimal. Ben-Gal et al. suggested simple and practical tips for patient care during the current outbreak, based on support networks, video consultation, use of telemedicine and telerehabilitation, enabling “clean” pathways throughout the hospital facilities, and providing pre-prepared packs for the required materials [33]. The HM3 system has the properties for unilateral data transfer from the patient’s home to a medical setting but due to cybersecurity issues the data transfer properties of the device are disabled. These security issues should be resolved. Furthermore, a bi-directional communication between the patient and the LVAD clinic should be safely constructed for the sake of preventing unnecessary travel and exposure of this vulnerable and frail population to a health care institution. A multi-disciplinary healthcare team including LVAD coordinator, nurse and physician should be prepared and trained for this purpose. For the moment, patients should be advised to record vital signs, LVAD parameters, weight, INR values and signs of COVID-19 infection (i.e., fever, cough, myalgia, loss of smell, and dyspnea). The LVAD coordinator should record device parameters, and the patient is encouraged to send the driveline exit site picture. Teleconsultation for LVAD patients is critical particularly during this pandemic in order to recognize early symptoms of acute infection. Patients should be instructed to pay attention to more specific symptoms such as loss of smell that may suggest COVID-19 infection rather than heart failure exacerbation. All LVAD implants patients should be encouraged for vaccination to minimize the risk of infection and to enable more reliable follow-up. For non-vaccinated individuals, serial reverse transcription polymerase chain reaction (RT-PCR) tests is reasonable. In summary, various factors contribute to increased mortality among patients with ventricular assist device (LVAD) during the current COVID-19 pandemic. Direct effects of the acute infection may compromise right ventricular (RV) and device function through multiple hemodynamic changes as part of the cytokine storm and sepsis. If acute respiratory distress syndrome (ARDS) develops, the patient may require prolonged mechanical ventilation (MV). Moreover, disturbances in coagulation pathways that accompany the course of COVID-19 have several implications on LVAD patients since their outcomes also depend on accurate hemocompatibility profile. Social deprivation and depression among elderly patients may lead to loss to follow-up and inappropriate management that also contribute to increased morbidity and mortality (Figure 1).

## 7. Conclusions

The current COVID-19 pandemic presents several diagnostic and management challenges for LVAD patients. Clinicians should be aware of the potential complications and the hemodynamic, mechanical and psychologic implications of this disease. Patients with LVAD and suspected COVID-19 should be managed in specialized centers with qualified teams.
